# Reducing the global burden of type 2 diabetes by improving the quality of staple foods: The Global Nutrition and Epidemiologic Transition Initiative

**DOI:** 10.1186/s12992-015-0109-9

**Published:** 2015-06-04

**Authors:** Josiemer Mattei, Vasanti Malik, Nicole M. Wedick, Frank B. Hu, Donna Spiegelman, Walter C. Willett, Hannia Campos, Global Nutrition Epidemiologic Transition Initiative

**Affiliations:** Department of Nutrition, Harvard T.H. Chan School of Public Health, Boston, MA 02115 USA; Department of Epidemiology, Harvard T.H. Chan School of Public Health, Boston, MA 02115 USA; Department of Biostatistics, Harvard T.H. Chan School of Public Health, Boston, MA 02115 USA

**Keywords:** Type 2 diabetes, Global health, Nutrition, Nutrition transition, Carbohydrate quality

## Abstract

**Background:**

The prevalence of type 2 diabetes has been reaching epidemic proportions across the globe, affecting low/middle-income and developed countries. Two main contributors to this burden are the reduction in mortality from infectious conditions and concomitant negative changes in lifestyles, including diet. We aimed to depict the current state of type 2 diabetes worldwide in light of the undergoing epidemiologic and nutrition transition, and to posit that a key factor in the nutrition transition has been the shift in the type and processing of staple foods, from less processed traditional foods to highly refined and processed carbohydrate sources.

**Discussion:**

We showed data from 11 countries participating in the Global Nutrition and Epidemiologic Transition Initiative, a collaborative effort across countries at various stages of the nutrition-epidemiologic transition whose mission is to reduce diabetes by improving the quality of staple foods through culturally-appropriate interventions. We depicted the epidemiologic transition using demographic and mortality data from the World Health Organization, and the nutrition transition using data from the Food and Agriculture Organization food balance sheets. Main staple foods (maize, rice, wheat, pulses, and roots) differed by country, with most countries undergoing a shift in principal contributors to energy consumption from grains in the past 50 years. Notably, rice and wheat products accounted for over half of the contribution to energy consumption from staple grains, while the trends for contribution from roots and pulses generally decreased in most countries. Global Nutrition and Epidemiologic Transition Initiative countries with pilot data have documented key barriers and motivators to increase intake of high-quality staple foods.

**Summary:**

Global research efforts to identify and promote intake of culturally-acceptable high-quality staple foods could be crucial in preventing diabetes. These efforts may be valuable in shaping future research, community interventions, and public health and nutritional policies.

## Background

Type 2 diabetes is one of the world’s most prevalent, costly, and fatal chronic conditions [1]. As of 2013, approximately 382 million people worldwide had the disease, with 175 million cases undiagnosed [1]. These cases occurred at disproportionate levels in low- and middle-income countries (LMIC) [1, 2]. Recent reports illustrate steep increases in the global prevalence of type 2 diabetes over the past few decades, and these trends are projected to keep rising over the next 20–40 years [3, 4]. At the population level, two factors have been proposed as major reasons for the rapid increase in diabetes worldwide: an epidemiologic transition where communicable diseases have decreased as the major causes of death [5], and a concurrent nutrition transition characterized by increasingly unhealthy dietary habits, combined with lower levels of physical activity [6].

Several scholarly articles have illustrated the nutrition transition by describing the increase in the *quantity* of macronutrients, mainly fats and carbohydrates, consumed per capita in most countries [6, 7]. On the other hand, the *quality* of the diet, specifically of carbohydrates, has significantly and dramatically changed in the past 50 years [7–10], yet has received less attention in the literature. Thus, there are few examples of how the change in the quality of major staple sources of carbohydrates, along with increased quantity, has been a driving force in the rapid diabetes epidemic within the context of the epidemiologic transition. As this is an imperative worldwide public health concern, substantial effort at the global level is needed to understand the contributors to diabetes and find appropriate solutions for it. To this end, investigators from twelve sites around the world (Nigeria, Tanzania, Kenya, India, China, Malaysia, Brazil, Mexico, Costa Rica, Kuwait, Puerto Rico, and USA), representing various stages of economic development and the nutrition transition, have formed the Global Nutrition and Epidemiologic Transition Initiative (GNET) [11] to assess the carbohydrate quality of staple foods in each country, and to develop culturally-appropriate interventions that would improve such traditional diets with the overarching goal of preventing type 2 diabetes. For this report, we refer to ‘staple food’ as one consumed frequently and in sufficient quantities as to constitute the dominant part of the diet and supply a substantial proportion of energy and nutrient needs [12]. We focus on staple foods contributing to carbohydrate intake, which include rice, wheat, maize (corn), millet, sorghum, roots and tubers (potatoes, cassava, yams and taro), and legumes [12].

The goals of this article are to (1) depict the global health crisis of type 2 diabetes in light of the epidemiologic and nutrition transition, (2) posit that worsening carbohydrate quality of traditional staple foods is a major contributor to the diabetes epidemic, (3) exemplify how efforts from a global initiative for diet interventions may serve as a model for other culturally-appropriate programs, and (4) consider the implications of such an initiative on future research, interventions, public health policies and practices, and dietary recommendations.

To attain these goals, we used demographic data from the United Nations World Population Prospects 2012 [13], and 2008 mortality data reported by the World Health Organization (WHO) and the United States Centers for Disease Control and Prevention (CDC) [14, 15] to depict the epidemiologic transition. The global diabetes burden was illustrated with data from the International Diabetes Federation [1]. The nutrition transition was described using data from the Food and Agriculture Organization (FAO) Food Balance (supply) sheets [16]. In addition, we compiled the results from preliminary studies and current efforts conducted in GNET countries to describe reasons, preferences, and feasibility of consuming staple foods.

### The epidemiologic transition

The epidemiologic transition has been described as the “complex change in patterns of health and disease and on the interactions between these patterns and their demographic, economic and sociologic determinants and consequences” [5]. The drivers of the epidemiologic transition are multifactorial, but include social and economic growth, urbanization, and globalization of technologies and food production [17]. Higher gross national products and per capita incomes help generate resources that can help manage and control the overall burden of death, which in turn increases life expectancy at the population level.

**Fig. 1:**
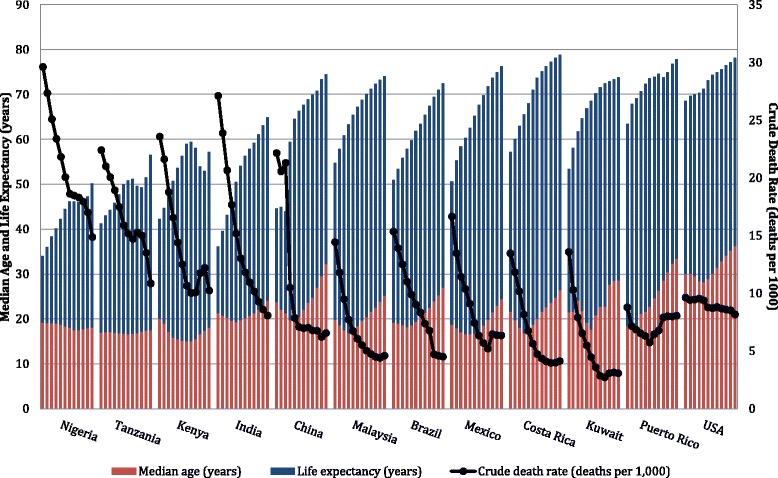
Epidemiologic transition in twelve countries, by 5-year period from 1950–2010. Data obtained from United Nations World Population Prospects: The 2012 Revision. Crude death rate reflects the number of deaths over a given period divided by the person-years lived by the population over that period. Life expectancy is the average number of years of life expected by a hypothetical cohort of individuals who would be subject during all their lives to the mortality rates of a given period. Median age is the age that divides the population in two parts of equal size. Tanzania includes Zanzibar. Data for China do not include Hong Kong and Macao, Special Administrative Regions (SAR) of China, and Taiwan Province of China. Malaysia includes Sabah and Sarawak

We depict the epidemiologic transition by contrasting the shift in mortality and population demographics from 1950 to 2010 (Fig. 1) [18]. The general trend has been a steep increase in life expectancy, alongside even steeper declines in crude overall death rates. In most countries, median age has increased slightly, suggesting an older demographic composition. However, distinct characteristics are observed for countries across stages of economic development. For example, in LMIC such as Nigeria, Tanzania, and Kenya, life expectancy remains low despite a dramatic decrease in the crude death rate. These countries also have a younger population based on median age. We classify these countries as being in ‘early transition’. India and China also show dramatic decreases in overall mortality, but with stronger economic growth and improvements in health care, their life expectancies and median age are higher than in lower income countries. Thus, we categorize India and China as countries ‘ongoing transition’, along with Malaysia, Brazil, and Mexico, which have also experienced increases in life expectancy over the same timeframe. Finally, Costa Rica, Kuwait and the US, including the territory of Puerto Rico, are considered ‘transitioned countries’. These higher income countries have a less steep increase in life expectancy, and the decrease in death rate has stalled or even reversed in recent years.

**Fig. 2:**
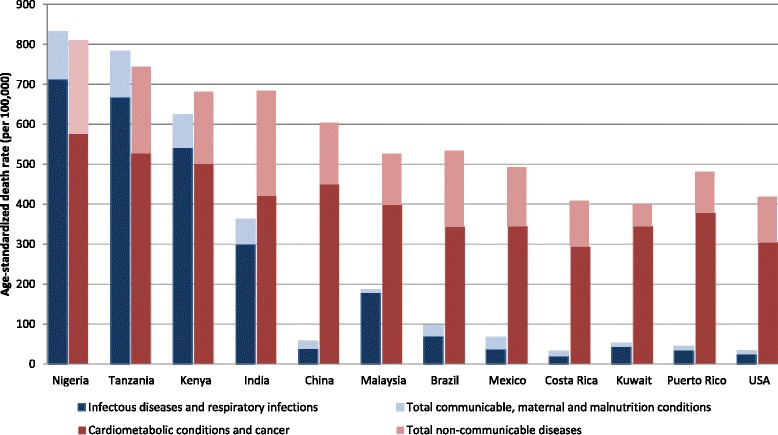
Age-standardized death rate by cause of death in twelve countries, 2008. Data obtained from WHO Global Burden of Disease Death Estimates, 2008. Cause-specific death rates were age-standardized to the WHO global standard population by applying age-specific death rates for the country to a global standard population. Mortality estimates are based on analysis of latest available national information on levels of mortality and cause distributions as at the end of 2010 together with latest available information from WHO programs, IARC and UNAIDS for specific causes of public health importance. Cause of death categories and their definitions were defined using the International Classification of Diseases, Tenth Revision (ICD-10). Cardiometabolic conditions and cancer includes malignant and other neoplasms, diabetes mellitus, endocrine disorders, and cardiovascular diseases. Total non-communicable diseases additionally include diseases in sense organ, respiratory (non-infectious), digestive, genitourinary, skin and musculoskeletal, as well as congenital anomalies, oral conditions and neuropsychiatric conditions Data for Puerto Rico is from 2007, obtained from the Centers for Disease Control and Prevention, National Vital Statistics Reports Final Data for 2007. Population used for computing death rates are postcensal estimates based on the 2000 census estimated as of July 1, 2007. Numbers after causes of death are categories of the International Classification of Diseases, Tenth Revision (ICD–10). Infectious diseases include influenza and pneumonia, and HIV. Total communicable diseases additionally include infant deaths (exclusive of fetal deaths). Cardiometabolic conditions and cancer include diseases of the heart, essential hypertensive disease, cerebrovascular diseases, diabetes, and malignant neoplasms. Total non-communicable diseases additionally include Alzheimer’s disease, chronic lower respiratory diseases, chronic liver disease and cirrhosis, nephritis, nephrotic syndrome and nephrosis, and Parkinson's disease. Causes of deaths included for Puerto Rico differ from those for the other counties, thus caution should be made when comparing death rates

The epidemiologic transition also involves shifts in the causes of morbidity and mortality [5], with a general trend for communicable, maternal and malnutrition conditions being gradually replaced by chronic, non-communicable diseases (NCD) as the main cause of death, which becomes more pronounced as the transition progresses. The shift in cause of death is partly driven by a transition in the types of risk factors, with physical inactivity and overconsumption of energy and energy-dense (nutrient-poor) foods as key contributors [19]. To illustrate this, we show the uneven distribution between 2008 age-standardized death rates of total communicable, maternal and malnutrition conditions (and specifically for infectious diseases and respiratory infections) and total NCD (and specifically for cardiometabolic conditions and cancer) in GNET countries, according to the stage of transition (Fig. 2) [14, 15].

Early-transition countries are characterized by considerably high burdens of both communicable diseases and NCD. While infectious and respiratory diseases tend to comprise the majority of communicable diseases, these countries also have to manage deaths from maternal and malnutrition conditions (pregnancy- and childbirth-related, and nutrient deficiencies, as classified by WHO [20]). Countries in ongoing transition show much lower death rates from total communicable diseases, which are far exceeded by the number of deaths from NCD. The malnutrition experienced during fetal or early development and childhood may predispose individuals in these countries to eventual NCD [21]; and this risk becomes exacerbated with overconsumption of energy and energy-dense (nutrient-poor) foods in adulthood as these populations gradually adopt unhealthy lifestyle patterns [22, 23]. Inadequacies in health care resources and health delivery infrastructure in these countries also fuel the high NCD death rates. These inadequacies may include limited availability of (or access to) affordable preventative care or treatment regimens that could help manage risk factors of NCD, as well as of sufficient or well-equipped clinical facilities and staff [24, 25].

As we move into transitioned countries, the death rates from communicable diseases become much lower, but the burden from NCD prevails, especially cardiometabolic conditions and cancer. Socioeconomic progress has facilitated efforts in controlling malnutrition and infectious diseases as individuals have more economic resources to afford sufficient foods, and countries can provide food assistance programs and infectious diseases prevention and control measures for their residents. But socioeconomic progress has also enabled the costly epidemic of NCD in several ways, such as boosting urbanization (which is related to unhealthy lifestyles) and globalization and mass production of food items (which may be of lower quality). Type 2 diabetes deserves to be singled out because of its impact and role as a harbinger of other complications or NCD with similar underlying metabolic pathology.

### Global burden of diabetes

The rise in prevalence of type 2 diabetes at the global level has been well documented, particularly in south Asia, Latin America and the Caribbean, central Asia, north Africa, and the Middle East [3]. Projections indicate that diabetes will reach pandemic levels by 2030, with the most notable increases in LMIC countries [4, 26]. Most of these countries are also in early or ongoing transition.

**Fig. 3:**
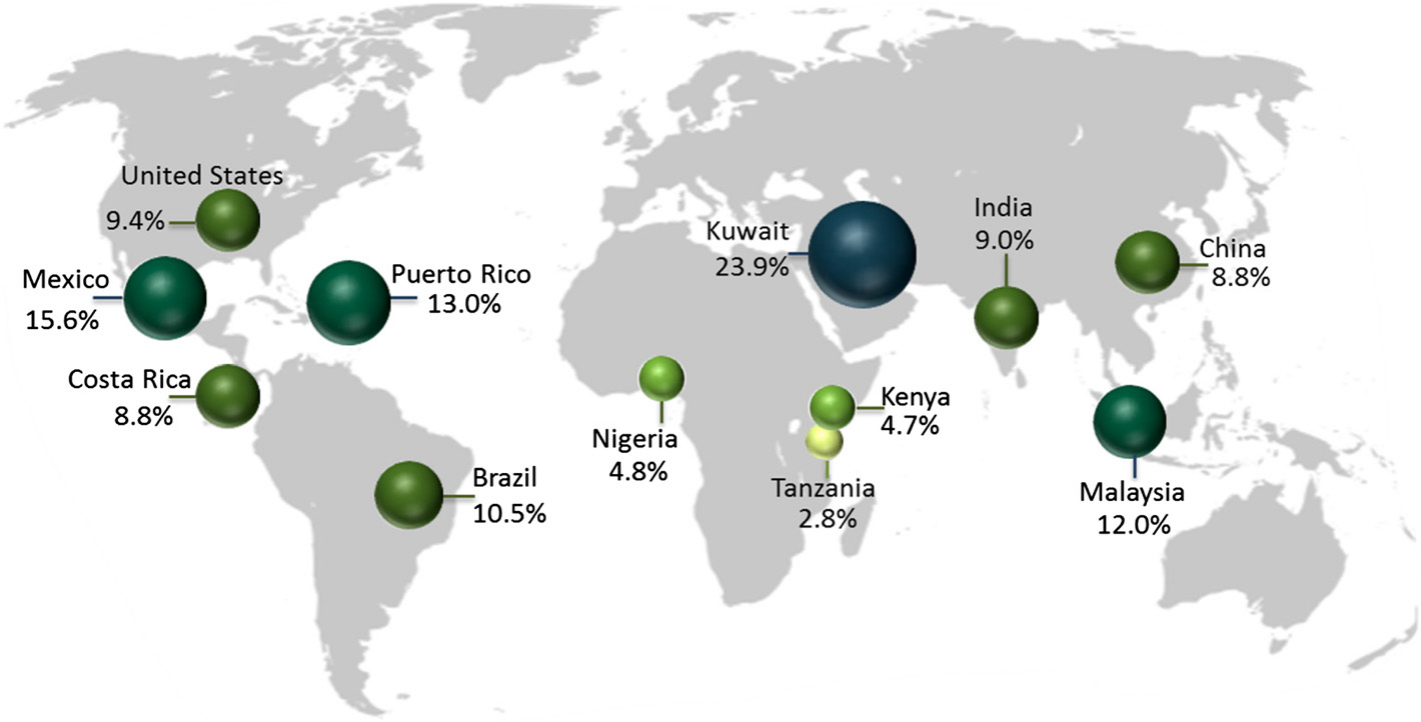
Prevalence of diabetes in twelve countries for the 20–79 age group, 2011. Data obtained from the International Diabetes Federation: Diabetes Atlas, 2012. The data are the comparative prevalence of diabetes, calculated according to the WHO standard, in the 20–79 age group

For the sites participating in GNET, the 2011 prevalence of diabetes ranged from 3 to 24 % (Fig. 3) according to stage of transition. While the prevalence remains low in early transition countries (3–5 %), the region will have one of the greatest relative increases in the next 15 years (161 %) [26], and it also has the highest percent (78 %) of undiagnosed diabetes cases [27]. Because diabetes and its comorbidities tend to predispose to some infectious conditions [28], the rise in diabetes in these early transition countries is even more alarming as they are still managing the burden of infectious diseases.

For ongoing transition countries, the prevalence of diabetes ranges from 9 to 16 % (Fig. 3). While we report a diabetes prevalence nearing 9 % for China, a new study estimated that 12 % of the population has diabetes and an additional 50 % has prediabetes [29]. China and India pose a distinctive situation, as their booming populations, economic growth, and urbanization rates, put them on the trajectory for a substantial increase in diabetes [26, 30–32]. By 2030, these two countries will remain as the two top countries with the highest absolute number of diabetes cases, increasing to nearly 80 million estimated for India (from 32 million in 2000), and to 42 million for China (from 21 million in 2000) [26]. Of concern, Asians tend to develop diabetes at a younger age (the prevalence of diabetes among 20–59 y/o adults is higher in Asian countries) and at a lower body weight compared to Western populations [33]. Another contributing factor is that many Asians have experienced considerable adversity, including malnutrition, during fetal and early life development [33], and these stressors may translate into genetic susceptibility to disease manifestation in adulthood through epigenetic mechanisms [33, 21].

Among ongoing transition countries in Latin America, Brazil is projected to have the highest prevalence of diabetes cases in the region (11 %) by 2030 [26]. Other projections list Mexico as the Latin American country with the highest diabetes prevalence (15 %) by 2035 [34]. This is likely, given a recent report from the United Nations placing Mexico as the world’s most obese country, with nearly 33 % of adults being obese [35].

Compared to other transitioned countries, Costa Rica has a relatively low diabetes prevalence, yet metabolic control among diabetes patients has been shown to reflect the measures observed in other industrialized countries [36]. The US is usually considered the benchmark of westernization and epidemiologic transition, and the prevalence of diabetes in this country is 9.3 %, but disparities among racial and regional subgroups have been reported [37–40]. Specifically, the US territory of Puerto Rico, which is ethnically a Latino population, has the highest diabetes prevalence in the US, with 13 % of the population having diagnosed diabetes [41]. Surveys conducted in the island point to even higher rates, especially in the western region, and among overweight/obese individuals in the metropolitan area [42, 43].

Still, among transitioned countries participating in GNET, Kuwait has the highest diabetes prevalence, with nearly a quarter of the population having the disease as of 2011. Kuwait has experienced tremendously fast economic growth, accompanied by adoption of unhealthy lifestyles since the end of the Gulf War in the early 1990s, likely contributing to the rapid increase of diabetes cases. Diabetes in the Kuwaiti population seems to start at a relatively young age, and with the ongoing increases in obesity (in 2010, approximately a third were overweight and 43 % were considered obese [44]), the mounting trends in diabetes in this country are of high concern [45, 46]. Of note, the rates of diabetes in Kuwait and many LMIC countries have not been standardized for age, which underestimates the reported prevalence and future burden of diabetes, because these countries generally have younger populations than developed countries.

### The case for improving carbohydrate quality for diabetes prevention

A prevailing indicator of carbohydrate quality is the glycemic index (GI), which ranks carbohydrate-rich foods according to their postprandial glycemic response relative to a reference carbohydrate source [47]. Foods and beverages with a high GI are hypothesized to contribute to the development of type 2 diabetes by rapidly increasing postprandial glucose concentrations and insulin demand, or postprandial free fatty acids that trigger insulin resistance [48, 49]. The amount of carbohydrate consumed also has an effect on postprandial glycemic and insulin responses. To reflect both carbohydrate quality and quantity, the measure of glycemic load (GL) estimates the product of the GI value of a given food multiplied by its carbohydrate content per serving [50].

The majority of observational studies suggest a positive association between GI or GL (slightly weaker for GL) and risk of type 2 diabetes [50–55]. Findings from clinical trials support these observations by showing improvements in markers of glycemic control on low GI/GL diets [56, 57]. Foods with a low GI or GL are generally rich in dietary fiber; which has been shown to have beneficial effects on diabetes risk and insulin sensitivity [58–60]. Whole grains are a major source of fiber in the diet and tend to have low GI values. In contrast, refined grains lack most of the germ and bran, which are removed during milling, resulting in the loss of numerous health-conferring constituents such as fiber, vitamins, minerals, lignans, resistant starch, phenolic compounds and phytochemicals [61]. Indeed, several large observational studies show strong inverse associations between intake of whole grains and risk of diabetes, with a range of 21-32 % risk reduction [62–65].

Studies looking at specific whole grain foods have reported similar findings. For example, substituting 50 g/d intake of white rice (a refined grain) with the same amount of brown rice (a whole grain) was associated with a 16 % lower risk of diabetes, and the same replacement with overall whole grains was associated with a 36 % lower risk [62]. An increase in a serving per day of white rice alone may translate into 11 % increased risk of diabetes [66]. The protective relation with brown rice has been shown by others, as well as with whole grain bread and wheat bran [65]. Clinical trials measuring biomarkers of diabetes risk with intake of whole grains have reported mixed results. In general, short-term feeding trials support the notion that switching to a whole grain diet may improve insulin response [67, 68]. Large trials in free-living individuals have not replicated these results [69, 70], but these type of studies tend to have inaccurate measurements and some bias due to the inherent ‘noise’ of free-living conditions. By properly addressing such limitations, replacing consumption of refined staple foods, such as white rice, with whole grain sources at the population level is one of the diabetes-prevention strategies being explored in GNET countries.

Legumes are another source of carbohydrate with high dietary fiber content, numerous phytochemicals, and low GI that are being considered in GNET countries. A cross-sectional study in Costa Rican adults found that increasing the ratio of beans to white rice, and limiting the intake of white rice by substituting beans, was associated with better levels of cardiometabolic risk factors [19]. Intervention studies have shown improvements in glycemic control after increasing the intake of legumes [71, 72]. Jenkins *et al.* found that incorporating legumes as part of a low GI diet reduced glycosylated hemoglobin, blood pressure, heart rate and risk of coronary heart disease, in patients with diabetes [73]. These findings reinforce the notion of promoting or reintroducing intake of legumes in the diets of countries that have undergone or are undergoing nutrition transition.

Finally, the evidence is scarce for the effect on diabetes risk of other staple carbohydrate sources, such as corn, roots, and tubers, as well as minor grains such as quinoa, barley, millet, and sorghum. Consumption of potatoes, which have glycemic properties equivalent to refined grains, has been associated with higher risk of type 2 diabetes [74]. A small intervention study among healthy non-obese adults that evaluated the GI of five mixed meals, some of which included plantains, corn, yams, and cassava, found that the GI of these meals were all similarly low [75]. Ancient grains (amaranth, barley, millet, quinoa, sorghum, spelt, whole-wheat couscous) have higher fiber content than white rice [76]. Depending on the degree to which corn is processed, it may either provide sufficient fiber to lower postprandial glycemic and insulinemic responses [77], or it may have potential detrimental effects on glycemic control and diabetes when processed into refined corn flour, corn syrup and high fructose corn syrup [78, 79], one of the main sweeteners used globally. Minimally-processed, whole kernel maize may be an appropriate alternative food in some cultures. Many of these carbohydrate sources (i.e., legumes, whole or partly-milled grains, yam, taro, plantains, some types of corn) have a high percent of resistant starch, for which there is some evidence of lower postprandial glucose, insulin and lipid levels [80], depending on the source and cooking process. More research on the metabolic effects of traditional meals that include these foods is warranted.

Although sugar sweetened beverages (SSB) are not considered a staple food, they are becoming a major contributor to the carbohydrate content of the diet in the form of empty calories and added sugars, particularly in developing countries [24]. In fact, sugary beverages (i.e.: soda, fruit drinks, sports drinks) are the top contributor of added sugars in the US diet along with desserts, snacks, sweets, and candy [81, 82]; these are foods that are widely consumed worldwide as well. Intake of SSB has been consistently associated with weight gain and risk of diabetes [83]. Interventions aiming to improve overall diet quality in global populations should thus also consider healthier beverage options.

### The nutrition transition: the concurrent downshift in carbohydrate quality

A major contributor to the epidemiologic transition has been the nutrition transition, a shift in food availability and consumption patterns from traditional minimally-processed diets to highly-processed unhealthy low-quality diets [84, 85]. The factors driving changes in food choices are manifold and complex, but it has been posited that rapid increases in country and per capita income and food purchasing power, changes to the food system and environment brought about by globalization and urbanization, expansion in global food trade and marketing, and cheaper prices of low quality foods, have all contributed [84, 85].

Dietary shifts have varied between and within countries, but some broad themes are apparent; for example, the increase in intake of fat and animal products [6]. While this implies a decrease in the *quantity* of carbohydrate sources, global reports suggest a worsening in the *quality* of carbohydrate sources in the diet, especially from staple foods, with a shift in intake from coarse whole grains or tubers to highly refined carbohydrates and added sugars [8–10, 86, 87, 25]. Yet, most of the literature has focused on changes in quantity of macronutrients rather than on changes in the sources of staple carbohydrate-rich foods that have occurred during the nutrition transition.

**Fig. 4:**
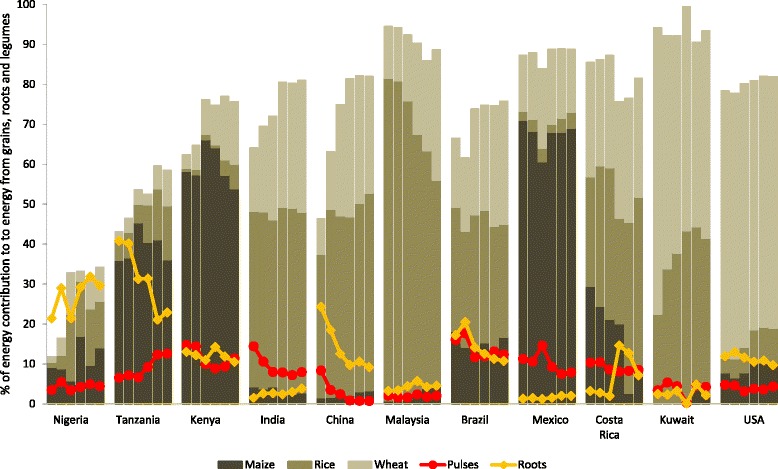
Contribution to energy consumption from grains, roots and pulses, for main staple carbohydrate sources, by 10-year period from 1961–2001 and 2009. Data obtained from the FAO Statistics Division: Food Balance (Supply) Data. Dietary energy consumption per person refers to the amount of food in kilocalories per day available for each individual in the total population during the reference period. Caloric content is derived by applying the appropriate food composition factors to the quantities of the commodities. Per person supplies are derived from the total amount of food available for human consumption by dividing total calories by total population actually partaking of the food supplies during the reference period. Per person Figure represent only the average supply available for the population as a whole and do not necessarily indicate what is actually consumed by individuals, which may be lower depending on the magnitude of wastage and losses of food in the household. All food items include edible whole and milled commodity and the derived products. Cereals used for alcoholic beverages were excluded. Other grain contributions not depicted in the figure include sorghum, millet, rye, barley, oats and buckwheat, quinoa, fonio, triticale, popcorn, and mixed grains. Pulses include all dry beans and peas (e.g.: chick peas, cow peas, pigeon peas, lentils). Roots include starchy roots and tubers (e.g.: cassava, plantains, potatoes, sweet potatoes, yams, yautía, and taro). Figure does not depict total carbohydrate contribution. Data not available for Puerto Rico

We depicted the shift in percent of contribution to energy consumption (denoting the amount of food in kilocalories per day available for each individual in the total population during the reference period) for the main staple carbohydrate sources (i.e., maize, rice, wheat, pulses, and roots and tubers) over the past 50 years in GNET countries (Fig. 4) [16]. The main staple food varied by country and region, but in general, diversity of carbohydrate sources increased, likely in part due to a rise in food trade with globalization. Overall, pulses and roots contribute less to carbohydrate intake than the other staple foods, and their intake appears to be decreasing over the years, compared to the stability or increase in the percent of energy from maize, rice, and wheat combined.

Early-transition countries derive most of the grain-based energy from maize, and while its contribution has remained fairly stable, rice and wheat products have been gaining popularity (Fig. 4). Consumption of pulses seems to be steady in the region, but traditional roots and tubers have taken a secondary role as staple foods. In India, China and Malaysia, rice has been steadily the main staple carbohydrate source, but wheat products have increased considerably. The contribution of the more wholesome pulses and roots and tubers in these countries is minor compared to rice and wheat.

Latin American and other transitioned countries show a more diverse range of the three main staple products (maize, rice, wheat), especially in recent years (Fig. 4). Pulses and roots and tubers do not represent a large contribution to intake despite them being considered traditional Latino foods. There was a spike in pulses in Mexico and in roots in Costa Rica in the 1980s, likely due to the economic crises experienced in those regions at that time, as these products tend to have lower consumer prices. However, the contribution of these foods quickly decreased in subsequent years. Finally, wheat remains the major staple carbohydrate source in two transitioned countries: Kuwait and USA, with rice increasing considerably in Kuwait.

Compiling data that could illustrate the nutrition transition was challenging due to the scarcity of historical data on carbohydrate consumption by source and level of processing by country, or of population-wide dietary surveillance data. We used FAO Food Balance data with the caveat that they include the commodity in its whole as well as milled form and we cannot distinguish the level of refinement of these foods. However, most of the grains in recent years were likely refined, as there is now more availability and demand for processed and pre-prepared foods, such as maize-, rice-, and wheat-based products [88]. For example, in the US, the major global exporter of wheat, the efficiency of milling wheat grain into flour (extraction rate) has steadily improved over the past 20 years [89], which may result in more availability of refined flour rather than the fiber-rich whole grain. Rice has been milled for centuries, but in India for example, as in many countries undergoing industrialization, the number of modern mills surpasses that of conventional ones, and these modern mills tend to be more efficient at removing the high-fiber husk and bran of the rice kernel [90]. Milled rice production worldwide is expected to increase by 35 million metric tons in the next ten years, but the area of harvested rice is projected to remain stable [91]. On the other hand, pulses, which include all dry beans and peas, and roots and tubers, tend to be consumed whole or with minimal industrial processing.

## Discussion

### Improving carbohydrate quality to prevent diabetes: examples from GNET countries

The eleven countries participating in GNET have the common goal of creating long-lasting, measurable improvements in the carbohydrate quality through sustainable, culturally-appropriate large dietary interventions. To reach these goals, the projects occur in four stages: (1) conducting dietary assessment to identify staple and alternative foods, as well as focus groups and taste tests to evaluate cultural attitudes and preferences for such foods; (2) testing the carbohydrate quality of foods and conducting pilot projects to determine the acceptability and feasibility of the foods and intervention settings in local communities; (3) implementing small-scale intervention studies to assess the effect of food substitution on intermediate markers of diabetes risk; and (4) developing a multicenter clinical trial with shared aims and measures but using country-specific foods and implementation strategies.

A main effort in GNET projects has been to assess the reasons for consumption of traditional and current staple carbohydrate sources. Eating is a basic biological need, and hunger and appetite tend to be the main drivers of eating; but people’s eating behaviors and food choices can be guided by many factors: sensorial (e.g. taste, appearance), availability, affordability, knowledge and skills about food or preparing foods, culture and customs, social (e.g. company, social norms) or psychological factors (e.g. stress, cravings), as well as personal attitudes (e.g. health beliefs) [92–94]. Learning about the reasons and perceptions for staple food choices at the population or community level may help tailor interventions and programs to encourage intake of healthy staples (or to reduce unhealthy ones) by taking advantage of the motivators while offsetting barriers. For example, the favorable nutrient-to-price ratio of cereals (whole grain) and beans [95] may be promoted as an affordable strategy to increase their purchase; or the preference (taste perception) for white bread over whole wheat bread (despite reasonable availability and knowledge of health benefits) may be compensated by altering its taste-affecting ingredients, processing, cost, or appearance [96–98]. To illustrate the value of understanding these factors, consumption of SSB has decreased after interventions reducing availability [99], increasing cost [100], or educating the population about their nutritional or health-related qualities [101]. GNET is exploring similar strategies within the context of a given country’s culture and stage of nutrition transition. The subsequent sections describe the formative assessments of traditional and current staple foods, and the dietary interventions informed by such, for each GNET site (summarized in Table 1).

**Table 1:** Staple foods contributing to carbohydrate intake, and summary of research activities in GNET countries

Stage of nutrition transition	Country	Traditional or potential high-quality staple foods	Current low-quality staple foods	Main findings and ongoing research activities
Early transition	Nigeria	• Fufu (pounded/mashed mixed meal of coarse cereals and roots)	• White rice	• White rice is current main staple food, and there are regional variations in staple food preferences
			• Fufu (mostly refined maize/cassava flour)	
				• Currently evaluating sensory attributes, barriers and motivators for replacing white rice with brown rice, and suggestions to promote intake
		• Roots and tubers		
Early transition	Tanzania	• Ugali (coarse maize flour mash)	• White rice	• Main reasons for increase in white rice included greater palatability; ease of storage; ease of preparation; variety of preparation methods; influence of Western dietary patterns
			• Ugali (refined maize flour)	
		• Millet	• Imported refined grains and cereals	
				• Whole grain ugalis and brown rice were highly rated for sensory perceptions; whole grain ugali was highly acceptable while brown rice was unpopular
Early transition	Kenya	• Ugali (coarse maize, millet or sorghum flour porridge)	• Ugali (refined maize)	• Maize remains primary staple, as well as rice and wheat products
			• Refined cereals and breads	
		• Beans, cassava, sweet potato	• White rice and wheat products	• Currently evaluating the glycemic index of potential high-quality alternative staple foods
Ongoing transition	India	• Hand-pounded rice (under milled)	• White rice	• White rice was preferred because of tradition, cooking quality, and appearance
			• Refined wheat products	
				• Health benefits of brown rice were unfamiliar
		• Coarse/whole cereals		• Willingness to switch to brown or undermilled rice, if affordable and had education on health benefits
		• Legumes and lentils		
		• Roots and tubers		• Suggestions to promote brown rice included advertising special recipes, celebrity endorsement, dispensing free samples, government-initiated education campaigns
				• Compared to white rice, brown rice improved 24- h glycemic and insulinemic response over 5 days using continuous glucose monitoring in overweight subjects; addition of legumes had no significant effect
				• Currently analyzing data from 3-month cross-over trial comparing brown to white rice meals on biomarkers in cafeteria setting
Ongoing transition	China	• Brown rice	• White rice	• Cultural barriers to accept brown rice were perception of rough texture, unpalatable taste, and higher price
		• Whole grains	• Refined wheat products	
			• SSB	
		• Legumes		• Promoting health benefits of brown rice could improve attitudes towards increasing its consumption
		• Roots and tubers		
				• Participants are willing to participate in brown rice intervention study
				• Intake of brown rice compared to white rice did not improve metabolic risk factors in a 16-week parallel-arm randomized intervention; some benefit on blood pressure in brown rice arm were observed among participants with diabetes
Ongoing transition	Malaysia	• Brown rice	• White rice	• Currently evaluating feasibility and acceptability of substituting brown rice for white rice, and barriers and motivators to consuming brown rice in 3 main ethnic groups
		• Legumes	• Refined wheat products	
		• Roots and tubers		
Ongoing transition	Brazil	• Brown rice	• White rice	• Currently identifying main contributors to carbohydrate and fiber intake
		• Legumes (beans)	• Refined flour breads	
			• SSB	
Ongoing transition	Mexico	• High-fiber nixtamalized commercial corn tortillas, or whole wheat tortillas.	• Commercial corn tortillas	• Currently analyzing data on consumption habits and attitudes towards beans
			• ‘Masas de maiz’ made with nixtamalized corn flour (bran removed)	
		• ‘Masas de maiz’ (corn dough) made from high-fiber nixtamalized corn flour		
			• Refined wheat flour bread	
			• White rice	
		• Legumes	• SSB	
		• Whole wheat bread		
		• Brown rice		
Transitioned	Costa Rica	• Brown rice	• White rice	• Brown rice was very unfamiliar
		• Legumes		• Tradition and family support were main drivers for intake of white rice
				• Consuming more white rice and fewer beans was engrained in the culture
				• Beans-to-white rice ratio of 1:1 was rated as most pleasant among 8 white or brown rice and beans preparations
				• Strategies to increase brown rice and bean intake included introducing them in childhood, promoting health benefits, lowering cost, increasing availability, masking unpleasant sensory qualities, and engaging women as agents of change
Transitioned	Kuwait	• Brown rice	• White rice	• Factors influencing consumption habits were taste, ease of preparation, and cost
		• Wheatberries (e.g. Jereesh, Harees)	• Refined grain (wheat) products	
				• Barriers to substituting refined grains with whole grains included unfamiliar taste, long cooking times, lack of cooking knowledge, cost, lack of availability
		• Legumes (lentils)		
		• Whole wheat bread		
				• Motivators to promote healthier consumption were awareness about health benefits, learning how to prepare whole grain meals, more availability, reasonable prices
Transitioned	Puerto Rico	• Legumes	• White rice	• Overall positive perceptions of legumes; main reasons for consumption of legumes were taste and nutrition
		• Brown rice	• White bread	
		• Roots and tubers	• Cold breakfast cereals	
		• Whole wheat bread	• SSB	• Higher consumption of legumes among those with more positive opinions or more bean variety
		• High-fiber cereals		
				• Currently identifying other foods contributing to carbohydrate and fiber intake
				• Currently analyzing data from taste and focus group studies that assessed perceptions, motivators and barriers, and taste preferences for of four possible replacement foods
				• Currently analyzing data from 3-week pilot to determine compliance and acceptability of possible replacement foods

*SSB* Sugar-sweetened beverages

### Countries in early transition

#### Nigeria

The major dietary carbohydrate sources in Nigeria have traditionally included roots and tubers, usually prepared as *fufu*, which is a pounded/mashed mixed meal of starchy vegetables consumed with a variety of side dishes. *Fufu* is usually made with cassava, yam, plantain, corn, wheat, or rice [102]. Cassava is also processed into a flour-like powder [103]. There is evidence that Nigerians are now consuming more rice, concurrent with a greater influence of Western lifestyle habits. In collaboration with GNET investigators from the Institute of Human Virology in Abuja, Nigeria, we completed a cross-sectional dietary assessment among urban Nigerian adults which showed that parboiled long grain white rice, typically imported from Asia or the US, is the now most commonly consumed carbohydrate [102]. Intake of white rice has shifted from being consumed mostly on the weekends and/or for celebratory purposes 40 years ago, to nearly daily consumption mostly on weekdays, with usually 3 servings per meal. Other traditional foods (*fufu*) remain common, but variation exists among the country’s regions for staple preferences.

#### Tanzania

In major parts of the country, the primary traditional carbohydrate food is *ugali,* a maize porridge often consumed at least twice per day, especially in rural areas [104]. Rice seems to be a staple food in urban settings [104]. Increases in imported cereals rather than use of traditional grains such as millet, as well as proliferation of processed and packaged food in urban areas [105], could be fueling the transition. A survey conducted by GNET colleagues at Muhimbili University (Dar es Salaam) found that the reasons identified for the increasing popularity of rice included greater palatability relative to other traditional staples, ease of storage, ease of preparation, variety of methods of preparation, and influence of Western dietary patterns, particularly among the young and middle class [106]. We recently published findings from preliminary taste tests and focus group discussions among overweight adults in two regions in Tanzania (Dar es Salaam and Morogoro), that evaluated the preferences, acceptability, and tolerability of whole grain staple foods [107, 108]. We found that all of the test foods (i.e., unrefined maize and sorghum *ugalis* and brown rice) were highly rated for smell, taste, color, appearance and texture. Refined maize *ugali* was preferred, but consumption of unrefined whole grain *ugali* was highly acceptable. Brown rice was unfamiliar and unpopular among participants, but nearly all were willing to participate in a future dietary intervention with whole grain alternative carbohydrate staples.

#### Kenya

Food habits in Kenya that were engrained as part of tradition have been rapidly changing with social development and modern lifestyle [109]. As in Tanzania, the main staple food is *ugali*, which in Kenya consists of a boiled-steamed mash made with maize flour, and sometimes with millet or sorghum flour, which are local grains [109]. Current staple foods are generally not as nutritious as the traditional ones unless they have been fortified [109]. Using food purchasing data collected from over 13,000 households, the Kenya Integrated Household Budget Survey indicated that cereals (namely maize) and breads are now the most important food groups [110].

GNET investigators at the University of Nairobi, Kenya are currently ascertaining the eating habits (e.g., frequency and amount, preparation and cooking methods) related to the major carbohydrate sources. In addition, we are evaluating the GI of several of these foods as a paucity of such data exists. Preliminary findings indicate that maize remains the primary staple, followed by beans, cassava and sweet potatoes. Similar to other African nations, we are noticing a shift from these foods towards more rice and wheat consumption. After we assess the current dietary patterns regarding carbohydrate consumption, we will then conduct focus group discussions to assess the acceptability of whole grain foods as substitutes for refined staple foods.

### Countries in ongoing transition

#### India

The nutrition transition in India is partly defined by sharp socioeconomic disparities within the country [111]. India is vast, and its dietary habits vary by region, but overall, where healthy foods such as coarse cereals, pulses, fruits and vegetables once dominated [112], processed rice and wheat products and meats now abound. Our GNET work is based in Chennai, India, in collaboration with researchers from the Madras Diabetes Research Foundation (MDRF). A previous study among adults in Chennai showed that refined cereals contributed 46 % of total energy intake and 65 % of the total daily carbohydrates [113]. Polished, refined white rice (parboiled) was the most frequently consumed cereal, while other traditional whole cereals, such as millet, as well as pulses and tubers, contributed lesser amounts to total energy. Thus, the work conducted at MDRF has focused on investigating the feasibility and cultural acceptability of substituting brown rice and under-milled rice, for polished white rice, using focus groups and taste tests among healthy-weight and overweight adults. Findings indicated that although most participants preferred white rice, they were willing to switch to brown or undermilled rice provided that it was affordable and they received education regarding its health benefits [114]. Additional suggestions to promote brown rice included advertising special recipes, celebrity endorsement, dispensing free samples and government-initiated education campaigns [115]. Tradition, cooking quality and appearance determined the specific form of rice that people purchased and consumed, while awareness of the health benefits of brown rice was not a major factor [114, 115].

Subsequently, our team compared the effects of white rice, plain brown rice, or brown rice with legumes on glycemic and insulinemic responses over 5 days on overweight subjects using continuous glucose monitoring. We showed that the brown rice menu significantly improved glycemic parameters compared to the standard white rice menu, with the addition of legumes not providing any significant improvement to brown rice alone [116]. We are currently implementing a 3-month randomized crossover trial comparing brown rice to white rice meals (breakfast and lunch, 6 days per week) on biomarkers of diabetes risk among adults at high risk for developing diabetes in a cafeteria setting at MDRF.

#### China

Similar to India, China is a highly-populated and vast country with many different dietary practices. In general, traditional diets were characterized by high intake of rice, whole grains, and vegetables, with little consumption of meats, wheat flour and maize/coarse grains; modern staple foods include refined rice or wheat products (buns/breads, cakes and sweets), animal products, processed foods, and SSB [117–120]. Intake of legumes, tubers and rice has decreased in both urban and rural areas [120]; white rice is still a staple food throughout the country. A recent study among Shanghai adults reported that 70 % of total carbohydrates came from white rice, followed by 17 % from refined wheat products [121].

Shanghai, China was the first site to join GNET with collaborators from the Institute for Nutritional Sciences, Shanghai Institutes for Biological Sciences, and the Chinese Academy of Sciences. An initial focus group and taste tests among middle-aged adults found that the main cultural barriers to the acceptance of brown rice were the perception of rough texture, unpalatable taste, and higher price [122]. Yet, most participants suggested that promoting the health benefits of brown rice could change societal attitudes towards it and nearly all participants were willing to participate in a future long-term brown rice intervention study.

Using this information, we completed a parallel-arm dietary intervention among adults with metabolic syndrome or diabetes randomized to brown rice or white rice for 16 weeks (lunch and dinner, 6 days per week). Cooked rice was provided at designated university cafeterias during the week and taken home for evening and Saturday meals. We observed that substituting brown rice for white rice did not lead to substantial improvements in metabolic risk factors although some statistically significant benefit on blood pressure was observed in the brown rice arm among participants with diabetes [123]. The results may have been limited by reduced statistical power, or by compensating modifications in diet and lifestyle among participants randomized to white rice. This finding prompted us to reconsider a crossover rather than parallel design in future studies, such that all participants would have the opportunity to experience both intervention arms.

#### Malaysia

Comprised by three major ethic groups (Malay, Indian, and Chinese), the dietary patterns in Malaysia reflect those of India and China, with a traditional intake of rice, vegetables, and starchy foods [124, 125]. Globalization has promoted the intake of energy-dense foods [125], and while traditional patterns have been maintained in Southeast Asia, more sugars and oils are being added to those traditional recipes [124]. Pulses and starchy roots (especially cassava) are still consumed, but they represent a minimal contribution to energy [124, 125]. Rice remains the main food staple, providing nearly a third of daily energy intake, in both rural and urban areas [124].

Recently, researchers from the Universiti Sains Malaysia, in Kelantan, Malaysia joined GNET to design a focus group study to evaluate the feasibility and acceptability of substituting brown rice for white rice, among all ethnic groups in the country. The study will identify perceived barriers to consuming brown rice and potential ways to increase consumption. The focus groups will be conducted in various regions of the country to capture the ethnic and cultural differences that may shape attitudes of rice intake. As with other sites, findings from the focus groups will help design the dietary intervention, which we intend to conduct among high-risk (overweight/obese) adults from the three major ethnic groups.

#### Brazil

Brazil is the most recent country to join GNET efforts. Investigators from the Universidade Federal do Rio Grande do Sul in Porto Alegre, Brazil are currently identifying which foods contribute most to carbohydrate and fiber intake among adults in this Southern region, as well as how strongly they are associated to metabolic outcomes. We will then assess the perceptions and preferences of various food preparations in their usual form along with healthier versions, as possible options for a diabetes-prevention dietary intervention. We posit that white rice and beans will be top contributors to carbohydrate intake. A national survey found that the most frequently consumed foods in Brazil were rice, beans, and bread, with a high prevalence of intake of SSB, especially in the Southeast region [126].

#### Mexico

Maize has been the staple grain in Mexico for centuries. Traditional corn-based foods include corn-based tortillas and *‘masas de maiz’*, a corn-based dough used as base for many typical foods that are then mixed with other ingredients, such as vegetables and legumes, into a meal. Corn flour is generally nixtamalized, a process on which a calcium hydroxide solution is added to the corn, and which alters the physical, chemical, and nutritional properties of the flour. The process also removes the corn kernel bran, thus total dietary fiber tends to decrease with higher concentration of the solution [127]. Moreover, total dietary fiber is lower in commercially-prepared nixtamalized corn flour than in flour made using traditional nixtamalization [128]. Refined wheat flour breads (‘*bolillo*’) and white rice are also commonly consumed. Increased purchases of refined carbohydrates and SSB have been replacing these traditional meals since the 1980’s [129, 130]. In 2012, beverages represented 17.5 and 19.0 % of the total daily energy intake per capita in children and adults, respectively [130]. Regional differences on staple food intake exist, as a study among pregnant women found that wheat tortillas and beans were mostly consumed in the Northwestern region but not in Mexico City [131].

With researchers from the Instituto Nacional de Salud Pública in Mexico City, we are exploring the consumption habits and attitudes towards beans with surveys implemented among adults, in an effort to understand how and why intake of this traditional food has changed. Using taste tests and focus group studies, the team will then evaluate possible high-quality carbohydrate alternatives such as high-fiber nixtamalized commercial corn tortillas or whole wheat tortillas, high fiber-nixtamalized corn flour products, bread made with whole wheat flour, or brown rice, as well as the possibility of re-introducing or increasing beans in the diet. Because of the excessive consumption of SSB in Mexico, the team is also considering evaluating the acceptability of healthy beverage alternatives in the focus groups.

### Transitioned countries

#### Costa Rica

As in most Latin American countries, the Costa Rican diet has been based predominantly on rice and legumes (namely beans) as staple foods for generations. However, there has been a shift in the amounts that each of those foods contribute to energy, with white rice representing the main source of energy [132, 133] but intake of beans declining, especially in urban areas [134].

In Costa Rica, we have documented that higher intake of white rice was associated with adverse levels of metabolic risk factors, while the opposite was noted for beans [19]. Moreover, a healthier cardio-metabolic profile was observed with increasing ratios of beans to white rice, or by substituting one serving of beans for one serving of white rice. Using this information, the GNET team at the Costa Rican Institute for Research and Education on Nutrition and Health (INCIENSA) at Tres Ríos have assessed the sensory perceptions of 8 traditional white or brown rice and beans preparations at different ratios, among middle-aged Costa Ricans. They coupled this with focus groups that aimed to identify barriers and motivators that could change current unhealthy dietary habits into healthier one. The investigators found that traditional habits and family support were the two main drivers for current consumption, and that consuming more white rice and fewer beans, as well as unfamiliarity with brown rice, were habits engrained within the Costa Rican culture [135]. The participants suggested that introducing brown rice and beans during childhood, disseminating health benefits, lowering the cost, increasing availability, masking unpleasant sensory characteristics, and engaging women as agents of change, were all possible strategies to increase their consumption. The preparation most rated as pleasant was the beans: rice 1:1 ratio, regardless of the type of rice. We are now quantitatively assessing how traditional cooking methods for rice and beans may influence consumption. For such, we are considering both the quantity and quality of ingredients added to the recipes of each food, in association with actual intake.

#### Kuwait

Dietary habits in Kuwait have dramatically changed in response to sudden economic growth. During times of austerity, foods consumed most widely included rice, dates, seafood, camel milk, and sheep and goat products [44]. After the discovery of oil in the region, the food supply became inundated with imported foods (comprising 85 % of the market) and fast-food outlets.

In Kuwait, we are working with researchers at the Dasman Diabetes Institute to identify high quality carbohydrate foods as substitutes for the main refined staple foods (white rice and white bread). We have conducted taste tests and a focus group study among overweight young and middle-aged adults from Kuwait city. A younger age group was included because diabetes risk appears to begin at a younger age in Kuwait [45, 46]. Potential substitution foods evaluated were brown rice and wheat berries (e.g. Jereesh, Harees) with and without lentils as replacements for white rice, and whole wheat bread as a replacement for white bread. Results from the focus groups illustrated that taste, ease of preparation and cost were among the factors that most influence consumption habits. Barriers to substituting refined grains with whole grains included unfamiliar taste, longer cooking times, lack of knowledge about cooking methods, cost and lack of availability. Participants suggested that increasing awareness about the health benefits of whole grains, learning how to prepare whole grain meals, increasing the availability and competitive prices of whole grain foods are potential motivators to promote consumption.

#### Puerto Rico

As a US-territory, Puerto Rico’s nutrition transition has been coupled to that of the mainland. During the first half of the 20th century, white rice and beans accounted for 47 % of total energy intake, with homegrown starchy roots and vegetables such as cassava, plantains, taro and ‘*yautía*’ (tannia/tannier/cocoyam) also commonly consumed [136, 137]. The second half of the century saw a decrease in legumes and root consumption and an increase in cereals [138]. A recent survey in the San Juan metro area documented consumption of main food groups consistent with a nutrition transition [139]. Similarly, a small study conducted in overweight/obese Puerto Rican adults showed high daily consumption of sweetened drinks (1.9 servings/day) and low intake of total dietary fiber (10.5 g/day) [140].

Researchers from the University of Puerto Rico Medical Sciences Campus in San Juan, Puerto Rico, conducted focus groups among obese non-diabetic adults to assess the perceptions, motivators and barriers for intake of beans, brown rice, SSB, and cold cereals. The team also conducted a 3-week pilot randomized intervention among overweight or obese adults without diabetes to determine compliance and acceptability of four possible replacement foods (brown rice, dry beans, high-fiber breakfast cereals, and coconut water), which were provided to participants and assessed repeatedly to help promote changes at home. The studies were preceded by blind taste tests of the four replacement foods. Analyses of these data are ongoing but preliminary results show that participants were generally receptive to incorporate brown rice and more dry beans in their diet.

To supplement this work, we conducted surveys to assess intake, perceptions, and dietary behaviors related to legume consumption in adults in San Juan. We found that most participants reported positive perceptions of legumes, deeming ‘taste’ and ‘nutritional value’ as the main reasons for consumption [141]. Additionally, a greater consumption of legumes was observed among those with a higher number of positive opinions towards beans, and those who consumed more legume varieties. In partnership with colleagues at Fundación de Investigación de Puerto Rico, we are now expanding assessment of other main staple sources of carbohydrates and perceptions towards such foods, and developing an intervention to assess how the healthy replacement foods affect biomarkers of diabetes.

### Summary

In this section, we identify areas of need as well as opportunities and implications of dietary studies for diabetes prevention through a global academic partnership (Table 2). We documented the simultaneous epidemiologic and nutrition transition, as illustrated with data from 11 countries across the economic development spectrum. The factors leading to these shifts are multiple and complex, but we proposed that declines in the quality of carbohydrates over time have contributed to the nutrition transition and thus to the increase in diabetes. We depicted the trends in contribution to energy consumption for the main staple carbohydrate sources in these countries, and highlighted the importance of representing changes in quantity of staple foods as percent contribution to energy rather than absolute numbers in order to show actual changes in the distribution of such staple foods across time.

**Table 2:** Areas of need, strategies, and recommendations for global collaborative studies on carbohydrate quality and diabetes prevention

Data and research needs:	
•	Systematic and recurring dietary assessment and surveillance at the global level
•	Standardized data collection on level of processing and refinement and consumption by type of carbohydrate source
•	Studies on the carbohydrate quality and the effect of roots, tubers, minor grains, and mixed meals on diabetes biomarkers
•	Evidence of whole grain effects using sustainable and cultural approaches in larger studies in free-living, community settings
Potential strategies for global studies and promoting high-quality foods:	
•	Conduct formative research to identify main foods, cultural attitudes, and dietary preferences in the specific population
•	Adapt the intervention using culturally-accepted foods and settings, as supported by evidence
•	Preserve cultural preferences for sensory qualities of foods
•	Harness people’s willingness to switch to healthy foods and interest in health benefits into high participation in dietary interventions and programs
•	Promote health benefits of high-quality staple foods (knowledge and skills that could help increase intake; mass media health promotion)
•	Consider cost-reducing strategies of the high-quality staple foods, such as subsidies or incentives
•	Consider cost increases or limiting the availability of low-quality staple foods, such as taxes or bans
•	Develop large-scale global changes in food marketing, trade, promotion, regulations and policies
Challenges, opportunities, and recommendations for conducting global partnerships:	
•	Challenges include limited advocacy, capacity and resources; coordinating multiple sites; navigating diverse social norms and policies; and securing international funding
•	Trans-disciplinary partnerships can help share ideas, advice, education, training, capacity-building, resources, expertise, and funding
•	Leverage existing global policy frameworks
•	Partner with similar initiatives as well as with national government agencies and community partners

#### Limitations and research needs

While compiling the data for this report, we noticed a considerable scarcity in comprehensive dietary surveillance data at the population level for most countries, especially LMIC, even when national and international agencies have thoroughly collected data on socio-demographic measures and morbidity and mortality, and there are global nutrition-related surveys (albeit limited) for women and children. Dietary assessment may be challenging in countries with limited resources and impeding social dynamics [109], yet stronger global and national efforts should be undertaken to document their population’s diet systematically so that we can identify main food sources and trends in dietary habits, and inform interventions and food policies accordingly.

One limitation is that the FAO Food Balance data does not distinguish the level of processing or refinement of individual food sources, and changes in carbohydrate quality were inferred from other non-systematic reports. Some studies have tracked expenditure of added sugars, sweeteners or fiber intake for countries for which such data may be available, but there is no availability of standardized time-trend data by level of processing or refinement of carbohydrate sources at the global level.

The evidence from both observational and intervention studies is sufficiently strong to support the notion that improvements in the quality of main staple foods could lower the risk of diabetes. Strategies for such may include substituting whole wheat staple foods for refined ones (i.e. brown for white rice, whole grain bread for white bread, or high-fiber corn or whole wheat tortillas for refined flour tortillas), or increasing the consumption of legumes. One limited area of research is the role of other staple carbohydrate sources, such as roots and tubers and minor (ancient) grains on biomarkers of diabetes and diabetes risk. Further studies should examine the metabolic functions, association with disease, and potential health effects of these foods, as they are potential healthy sources of carbohydrates that may be familiar, culturally acceptable, and locally produced in most countries.

Most clinical trials and interventions measuring biomarkers of diabetes with intake of whole grains have been small, short-term, and implemented in controlled settings (118), showing modest effects at best. While even small improvements in metabolic markers through a better carbohydrate quality diet could lead to cumulative long-term benefits, more evidence is needed from larger studies in free-living, community settings using sustainable and culturally-appropriate strategies.

#### Contribution of GNET’s work to global studies for promoting high-quality foods

The formative research conducted in GNET projects has been instrumental towards our goal of improving the quality of staple foods. For example, we have learned that main staple foods, food preparations, and eating habits differ by country. Cultural attitudes and preferences towards food choices and the setting for interventions may influence how each population responds to the dietary programs. Cultural context and norms can in fact impact food consumption [142]. And while the GNET brown rice intervention in China did not show significant improvements in diabetes biomarkers among participants with diabetes, except for a potential benefit on blood pressure, it helped us formulate stronger strategies for study design in future projects. It would be impractical to create general interventions with the same food and setting across all populations. Identifying key foods, cultural acceptance and attitudes, dietary preferences, and proper logistics for each setting in a multisite study, can shape a customized intervention that is more likely to succeed.

Nonetheless, there were shared strategies for improving carbohydrate quality as mentioned across studies from GNET countries, such as disseminating the health benefits and reducing the cost of the high-quality staple foods. Taste and other sensory aspects were deemed important by participants from all countries, suggesting that cultural preferences for food quality should be preserved. Notably, the majority of participants in the GNET pilot studies conducted so far have reported having some knowledge of the health benefits of the proposed replacement foods, as well as willingness to switch to such foods if it helped improve their health. This provides timely and strong support to conduct such dietary interventions with a high potential for success.

Common motivators for incorporating staple foods of high carbohydrate quality into the diet may provide feasible opportunities for global strategies for diabetes prevention. For example, the cost of whole grain staple foods may be reduced through lower consumer prices, governmental subsidies, or inclusion in food aid or supplementation programs. Price manipulation can influence the purchases of healthy versus unhealthy foods [143]. Agricultural incentives for production of local grains and legumes could also help reduce price or increase consumption [144, 145]. Moreover, intake of refined carbohydrate products may be curbed with programs that increase their price or decrease their availability, similar to the numerous policies concerning SSB [146]. Nutrition education and mass health promotion strategies targeted to the population – including specific recommendations on carbohydrate-rich staple foods in a country’s dietary guidelines – can have a measurable effect on healthy dietary habits [147, 148]. Other large-scale global strategies that have been proposed to improve diet quality include food advertising and promotion of healthy foods; regulating the availability of processed vs. healthy products in supermarkets; promoting local markets; improving food trade and investing regulations; stronger nutrition labeling; and regulating food marketing practices [142, 149, 7].

Several GNET countries are already implementing some of these strategies. For example, Mexico’s Ministry of Health is promoting a nationwide communication program with recommendations to consume water instead of SSB [150], along with a recently implemented tax on SSB [130]. Kuwait’s government subsidizes low prices for staple foods (polished white rice, lentils, sugar) [44] and other carbohydrate-rich foods such as flour and bread. Such policies could be expanded to include whole grain alternatives to a greater extent than refined versions. China is engaging in systematic multi-sector efforts for NCD-prevention with agricultural, health care, and government groups, although representation from the nutrition sector is limited [151].

#### Challenges, opportunities, and recommendations for global partnerships

Siegel and Narayan have noted that global action towards diabetes prevention is constrained by lack of global advocacy and insufficient partnerships, capacity and resources, among other limitations [152]. GNET has indeed faced some challenges, including the effort of coordinating multiple sites, translating shared documents, familiarizing with social norms and practices for proper cultural adaptation and study logistics, learning the diverse agricultural and nutrition policies of each country for appropriate translation of results, and navigating and securing international funding opportunities. Yet, an advantage of working in a collaborative global academic initiative such as GNET is the potential to overcome some of these constraints by sharing interdisciplinary research ideas, scientific breakthroughs, education and training, expertise, capacity building, resources, and even funding [153, 154], which is especially valuable for institutions lacking any of these factors. Our multi-disciplinary team includes experts in biostatistics, epidemiology, medicine, nutrition, biochemistry, and other fields, that have mutually improved research competency.

Crafting global policies, especially across the food supply chain, can have tremendous impact, but may take time, effort, and coordination to come to fruition. A valuable model to inform this process is the NOURISHING framework, a compilation of worldwide policy actions prepared by the World Cancer Research Fund International to help policymakers identify population-specific approaches as well as areas of need around food policies for promoting healthy diets and reducing obesity and NCD [155]. Finally, partnering with similar initiatives, such as those from the International Diabetes Federation and the United Nations [152], as well as with national government agencies and community partners, could further strengthen common goals towards global diabetes prevention.

## Abbreviations

CDC: Centers for Disease Control and Prevention
FAO: Food and Agriculture Organization
GNET: Global Nutrition Epidemiologic Transition Initiative
GI: Glycemic index
GL: Glycemic load
LMIC: Low- and middle-income countries
MDRF: Madras Diabetes Research Foundation
NCD: Non-communicable diseases
SSB: Sugar-sweetened beverages
WHO: World Health Organization
